# Genome-wide association for grain yield under rainfed conditions in historical wheat cultivars from Pakistan

**DOI:** 10.3389/fpls.2015.00743

**Published:** 2015-09-22

**Authors:** Qurat-ul Ain, Awais Rasheed, Alia Anwar, Tariq Mahmood, Muhammad Imtiaz, Tariq Mahmood, Xianchun Xia, Zhonghu He, Umar M. Quraishi

**Affiliations:** ^1^Molecular Plant Breeding, Department of Plant Sciences, Quaid-i-Azam UniversityIslamabad, Pakistan; ^2^National Wheat Improvement Center, Institute of Crop Science, Chinese Academy of Agricultural SciencesBeijing, China; ^3^International Maize and Wheat Improvement Center (CIMMYT), C/O Chinese Academy of Agricultural SciencesBeijing, China; ^4^Higher Education Commission, Research and DevelopmentIslamabad, Pakistan; ^5^International Maize and Wheat Improvement Center (CIMMYT), C/O National Agriculture Research CenterIslamabad, Pakistan

**Keywords:** spring bread wheat, genome-wide association studies (GWAS), marker trait association, single nucleotide polymorphism (SNP), 90K SNP assay, gene annotation, grain yield

## Abstract

Genome-wide association studies (GWAS) were undertaken to identify SNP markers associated with yield and yield-related traits in 123 Pakistani historical wheat cultivars evaluated during 2011–2014 seasons under rainfed field conditions. The population was genotyped by using high-density Illumina iSelect 90K single nucleotide polymorphism (SNP) assay, and finally 14,960 high quality SNPs were used in GWAS. Population structure examined using 1000 unlinked markers identified seven subpopulations (*K* = 7) that were representative of different breeding programs in Pakistan, in addition to local landraces. Forty four stable marker-trait associations (MTAs) with -log *p* > 4 were identified for nine yield-related traits. Nine multi-trait MTAs were found on chromosomes 1AL, 1BS, 2AL, 2BS, 2BL, 4BL, 5BL, 6AL, and 6BL, and those on 5BL and 6AL were stable across two seasons. Gene annotation and syntey identified that 14 trait-associated SNPs were linked to genes having significant importance in plant development. Favorable alleles for days to heading (DH), plant height (PH), thousand grain weight (TGW), and grain yield (GY) showed minor additive effects and their frequencies were slightly higher in cultivars released after 2000. However, no selection pressure on any favorable allele was identified. These genomic regions identified have historically contributed to achieve yield gains from 2.63 million tons in 1947 to 25.7 million tons in 2015. Future breeding strategies can be devised to initiate marker assisted breeding to accumulate these favorable alleles of SNPs associated with yield-related traits to increase grain yield. Additionally, *in silico* identification of 454-contigs corresponding to MTAs will facilitate fine mapping and subsequent cloning of candidate genes and functional marker development.

## Introduction

Historically, breeding has contributed to radical increases in cereal production, particularly by the use of dwarf genes and exploitation of heterosis. However, grain production must increase by 70% from present levels to meet projected world food requirements by 2050 (Ray et al., 2013). Wheat as one of the most important cereal crops, will become increasingly important for food security under climate change conditions, in addition to the shortage of water and other resources. Wheat varieties with high yield and an appropriate end-use quality is the primary objective of all breeding programs around the world. Grain yield (GY) is governed by numerous genes that interact with each other and with the environment (Quarrie et al., 2006). Total Grain yield is a complex trait contributed by multiple yield components, and each component is a quantitative trait controlled or affected by several genes (Zhang et al., 2010b). Thus, there must be detailed genetic dissection of the yield trait in order to manipulate each gene to greatest advantage.

Although breeding progress for improved GY has been achieved for irrigated environments (Langridge and Reynolds, 2015), much less gain has occurred in rainfed environment (Ray et al., 2012). The problem is more severe in drought-prone environments, and the yield gaps between high production areas and dryland agriculture are very large. Strategies to reduce this gap include the genetic improvements under drought conditions by identifying sources of traits associated with drought tolerance and subsequent introgression of genes underlying the target traits to locally adapted cultivars. The challenge for implementing such strategies in breeding programs is identification of the most suitable target traits in a time-efficient and cost-effective way for different drought scenarios (Richards et al., 2014). Recent advancements in high throughput genotyping and phenotyping have improved understanding of the physiological and molecular basis underlying complex traits including drought tolerance (Salekdeh et al., 2009; Langridge and Reynolds, 2015).

Absence of a completely sequenced reference genome in wheat has limited gene discovery in bread wheat in last decade and recent advancement in field of functional genomics have rendered a new push to breeders to achieve their goals (Pingault et al., 2015). But at present, the best alternate choice for wheat breeder is the use of high-density single nucleotide polymorphism (SNP) assays to define genomic regions associated with quantitative traits either in bi-parental mapping experiments or in genome-wide association studies (GWAS) (Bordes et al., 2014; Edae et al., 2014; Zanke et al., 2014b). Several studies have identified chromosomal regions involved in yield performance, either by QTL analysis using linkage maps (Zhang et al., 2010b; Bennett et al., 2012; Liu et al., 2012; Mir et al., 2012) or by association mapping based on linkage disequilibrium (LD) (Bordes et al., 2013, 2014; Lopes et al., 2014; Zanke et al., 2014a,b). However, much more work is needed due to the bottleneck arising from the lack of inconsistency among studies, and use of low density marker platforms in gene mapping studies. Recently, several medium to high density SNP assays have been developed based on their distribution across the genome, minor allele frequency (MAF) and inter-variant LD using various technological principles (Akhunov et al., 2009; Saintenac et al., 2011; Poland et al., 2012; Wang et al., 2014). GWAS in wheat is challenging due to the large genome, incomplete genome sequence, and polyploidy, those make it difficult to assign the markers to the highly similar homeologous chromosomes (Sukumaran and Yu, 2014). Therefore, a combined meta-genomic approach using comparative analyses of cereals and GWAS may provide an opportunity to accelerate identification of genes controlling quantitative traits in wheat (Quraishi et al., 2011).

Bread wheat, a staple food crop in Pakistan, is grown on 7.8 m ha, including 2.2 m ha under rainfed condition. CIMMYT germplasm with 1BL.1RS translocation and their derivatives has been used both under irrigated and rainfed environments. However, they even provided better adaptability in rainfed environments, and contributed to reduced yield penalties associated with water stress (Tahir et al., 2014). Thus, much more efforts are needed to dissect genetic mechanisms underpinning adaptation to drought conditions and to devise marker-based breeding strategies that involve marker-assisted selection or genome-wide selection to obtain the required genetic gains. The present study was designed to identify the stable genomic regions associated with adaptation to rainfed conditions in historical wheat cultivars of Pakistan. We used the 90K SNP assay for GWAS for yield related traits, and SNP-flanking sequences were then used to predict the functions of MTAs involved in high yielding Pakistani cultivars.

## Materials and methods

### Plant materials and phenotyping

The plant material was a collection of 123 Pakistan spring wheat cultivars adapted to irrigated, semi-arid, and arid climatic zones (Supplementary Table 1). The collection comprised four groups based on genetic background and time of release; 10 cultivars pre-dated the Green Revolution i.e., cultivated before 1965, 23 were Green Revolution era released during 1960–1979, 45 post Green Revolution released during 1980–2000, and 45 were modern cultivars released during 2000–2014. The seeds were obtained from the Plant Genetic Resources Institute, National Agricultural Research Centre, Islamabad, Pakistan. Field trials were performed at the National Agriculture Research Centre, Islamabad in 2011–12, 2012–13, and 2013–14 seasons using an alpha lattice design with three replications of 1 × 2 m^2^ plots. Soil samples were collected from four random sites of each field/year and soil parameters were recorded before planting. The soil had mean EC values of 0.30 dS m^−1^, 7.8 pH, 0.70% organic matter, 4.5 mg kg^−1^ available-P, 130 mg kg^−1^ available-K and textural class loam 13.

Nine phenotypic traits were evaluated, including days to heading (DH), days to maturity (DM), plant height (PH), spike number (SN) (spike number m^−2^), grain numbers per spike (GN), thousand grain weight (TGW), grain yield (GY), biological yield (BY), and harvest index (HI). DH were recorded on 50% spike emergence, and DM were recorded when 50% of spikes showed total loss of green color. PH was measured after physiological maturity by measuring the distance between the stem base and the top of the spike excluding awns. SN was scored in 1 m sample and then transformed to spike number per m^2^ (SN). GN was calculated from the mean of 30 randomly selected spikes in each plot. After harvest, TGW was measured by weighing duplicates of 500 kernels from each plot. Grain yield (GY) was determined as the weight of grain harvested per unit area (g m^−2^). BY was estimated as the weight of the above ground biomass for whole plots at 72 h after harvest, and then HI was calculated.

### Genotyping

Five seeds of each cultivar were planted in 5 cm diameter pots. Fresh leaf samples for DNA extraction were collected from 25-day old seedlings. DNA extraction was carried out according to the CIMMYT Molecular Genetics Manual (Dreisigacker et al., 2012). Subsequently, DNA samples (50–100 ng/μl per sample) in 96-well plate formats were sent to CapitalBio® genotyping facility in Beijing for genotyping with high-density illumina 90K infinium SNP array (Wang et al., 2014). PowerMarker v. 3.0 was used to estimate genetic similarities between wheat lines with a Dice coefficient based on the proportion of shared alleles (Liu et al., 2005). Genetic diversity at each locus was determined by polymorphism information content (PIC). The positions of SNP markers along chromosomes in terms of genetic distance (cM) were based on the 2015 wheat consensus genetic map (Wang et al., 2014). Markers were removed from the dataset if they were either monomorphic, showed more than 20% missing values, had ambiguous SNP calling or exhibited allele frequencies of less than 5% (minor alleles).

### Statistical analysis

#### Phenotypes

Phenotypic data were analyzed with XLSTAT software 2010. Analyses of variance (ANOVA) and co-efficients of correlation were calculated for all traits.

#### Population structure and kinship analysis

Population structure was estimated with 1000 unlinked SNP markers using STRUCTURE software 2.3.3, which implements a model-based Bayesian cluster analysis (Pritchard et al., 2000). The STRUCTURE procedure organizes population entries into n cluster to form a structure matrix (Q-matrix). A putative number of subpopulations ranging from *k* = 1 to 15 was assessed using 100,000 burn-in iterations followed by 500,000 recorded Markov-Chain iterations. To estimate the sampling variance (robustness) of inferred population structure, 10 independent runs were carried out for each k. K was estimated using an *ad-hoc* statistic ΔK based on the rate of change in log probability of data between successive values (Evanno et al., 2005; Quraishi et al., 2011). LD among markers was calculated using observed vs. expected allele frequencies of the markers in TASSEL v.5.0. Only mapped markers were used for LD calculation both for the entire panel and for model-based subgroups. The critical *r*^2^-value beyond which LD is due to true physical linkage was determined by taking the 95th percentile of the square root transformed *r*^2^ data of unlinked markers (Breseghello and Sorrells, 2006). The percentage of marker pairs significant at different critical *r*^2^-values (0.2 and 0.2641) and *P* < 0.001 was determined for each chromosome to compare the degree of LD among genes.

#### Association analysis

For best linear unbiased estimates, a mixed linear model (MLM) procedure was used, where the genotypes, replications, rows, and columns were considered as fixed in the model for each environment. MLM was also used to check statistically significant differences among the genotypes for each trait, whereas the Best Linear Unbiased Predictions (BLUPs) and variance components were acquired for all traits. The unified mixed model approach (Q+K model) of Yu and Buckler (2006) was applied to the data using TASSEL Standalone v.5.0 to estimate marker—phenotype associations. The already estimated population structure (Q) and relative kinship matrix (K) were used in a MLM to test marker trait associations. Both phenotypic and genotypic data were imputed and filtered to reduce the data tree. The association between genotypic and phenotypic data was analyzed using the kinship matrix in MLM to control background variation. In order to account for the combined effect of such relatedness factors, kinship, and Q matrix were included as covariates in the regression model that defined the correlation between genotype and phenotype in association analysis (Quraishi et al., 2011). False discovery rate (FDR) analyses were conducted using Q-VALUE in R (Storey and Tibshirani, 2003).

#### Gene annotation and synteny of MTA

The flanking sequences of SNPs associated with traits were obtained from the Wheat 90K SNP array database (Wang et al., 2014). Gene ontology (GO) was assessed using BLAST2GO v.3 software (https://www.blast2go.com/), and SNPs were further annotated using the TriAnnot Pipeline in the International Wheat Genome Sequencing Pipeline.

A chromosome-based draft sequence of the hexaploid bread wheat was recently published (The International Wheat Genome Sequencing Consortium (IWGSC), 2014). Ditelosomic stocks of Chinese Spring wheat were used to isolate, sequence, and assemble de novo each individual chromosome arm, except for 3B, which was isolated and sequenced as a complete chromosome. Markers sequences were masked for repeat elements using a repeat masked database (http://www.girinst.org/server/RepBase/). Sequences of SNP markers linked with yield traits (henceforth: MTA) were BLASTed against wheat contigs of using a described previously methodology (Salse et al., 2008, 2009; Bolot et al., 2009). Sequences of *Brachypodium distachyon*, rice, sorghum pseudomolecules (builds Brachypodium 1.2 from http://www.brachypodium.org, Osativa_120 from http://phytozome.jgi.doe.gov/pz/portal.html, and Sorghum 1.0 from http://genome.jgi-psf.org/Sorbi1/Sorbi1.info.html) were then aligned against the wheat contigs and simultaneously against the identified MTA of all the traits. The criteria for alignment were escalated by considering three parameters reported in Salse et al. (2009), that is, AL (aligned length of sum of all HSP), CIP (cumulative identity percentage), and CALP (cumulative alignment length percentage). These parameters were applied to all BLAST alignments in the study.

## Results

### Phenotypic evaluation

The planting sites were located in the rainfed agroclimatic zone with reliability on rainfall, with 400, 406, and 560 mm in 2011–12, 2012–2013, and 2013–2014 seasons, respectively. Release year of cultivars had significant impact on phenotypic variation (*P* < 0.01). ANOVA showed significant differences (*P* < 0.01) among genotypes for most of the traits (Table 1). DH was non-significant in 2011–12 and 2012–13 seasons, falling within a range of only 7 and 8 days, respectively. Average GY during the three seasons ranged from 445.3 to 1787.6 g m^−2^. DH in 2013–14 (107.9 days) was longer than in 2011–12 and 2012–13 (91.4 and 97.4 days, respectively). However, BY was lowest in the third year (691.2 g m^−2^) due to lack of rain at the early growth stage. PH was significantly higher in 2013–14 (93.3 cm), while TGW was higher in year 2012–13 (Table 1).

**Table 1:** **Basic statistics for nine yield components in historical wheat cultivars evaluated in 3 years**.

**Trait^a^**	**2011–12**		**2012–13**		**2013–14**	
	**Range**	**Mean**	**Range**	**Mean**	**Range**	**Mean**
DH (days)	88–95.7	91.5	94–101.7	97.5	101–132	108
DM (days)	124.3–139	133.3	130.3–145	139.3	134–165	140.9
PH (cm)	62–110	81.2	59–120	86.7	62–123	93.3
GpS	21–72	44.3	27–81	48.7	19–68	45.9
SN (m^−2^)	189–575	487.3	211–510	463.5	245–623	502.4
TGW (g)	16.4–51.5	35.5	19.9–49.9	31	17.4–51.6	33.4
GY (g m^−2^)	224.3–518.5	341	179.4–642.2	301.2	139–843.6	352
BY (g m^−2^)	523.2–1043.3	704.5	420.1–1229.8	775.5	321.5–1623.2	691.2
HI	0.1–0.38	0.3	0.1–0.4	0.2	0.1–0.4	0.29

a *DH, Days to heading; DM, Days to maturity; PH, Plant height; GpS, Grains per spike; SN, Spike density; TGW, Thousand grain weight; GY, Grain yield; BY, Biological yield; and HI, Harvest index*.

GY was positively correlated with TGW (*r* = 0.70), BY(*r* = 0.85), and HI (*r* = 0.73) and (Table 2 and Supplementary Figure 1). PH and GY were not significantly correlated (*r* = 0.14), but PH was significantly correlated with TGW (*r* = 0.35) and BY (*r* = 0.24). Other correlations were non-significant or significant with very small *r*-value.

**Table 2:** **Pearson's coeffients of correlation between yield components based on data averaged over years**.

**Trait^a^**	**DH**	**DM**	**PH**	**SN**	**GPS**	**TGW**	**GY**	**BY**
DM	**0.97**							
PH	−0.07	−0.09						
SN	0.2	0.20	−0.11					
GPS	0.22	0.18	−0.10	0.19				
TGW	−0.09	−0.13	**0.35**	0.14	0.12			
GY	−0.09	−0.09	0.14	**0.32**	0.18	**0.70**		
BY	0.05	0.06	**0.24**	**0.53**	**0.25**	**0.50**	**0.85**	
HI	−0.03	−0.06	0.20	0.08	0.13	**0.67**	**0.73**	**0.43**

*Values in bold are different from 0, P < 0.05*.

a *See the footnote of Table 1 for abbreviations*.

### Analysis of SNP markers

Among the 81,000 SNPs in the 90K infinium assay, 596 (0.7%) were monomorphic and 19,810 (23.9%) had poor quality, thus were excluded from the analysis. The remaining 60,594 (69.1%) were further reduced to 14,960 (18.4%) by eliminating markers with minor allele frequencies (MAF, ≤ 0.03) and minimum counts of 80%. SNP dataset can be requested for future research via email.

A genetic framework map of all 21 wheat chromosomes was constructed using the 14,960 polymorphic SNPs projected onto the consensus map (Wang et al., 2014), resulting in an average of 712 markers per chromosome. However, the marker density for the D-subgenome was relatively poor with an average of 484 markers per chromosome. In total, the polymorphic markers spaned over a genetic distance of 3705 cM, with an average density of 0.13 cM per marker.

### Population structure and kinship matrix

**Figure 1:**
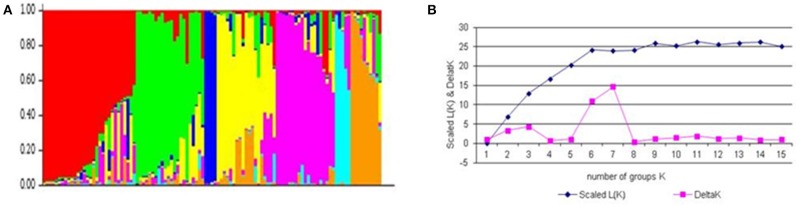
**Population structure of association mapping pannel based on unlinked SNP marker**. **(A)** Plot of the scaled average logarithm of the probability of data likelihood [LnP (D)] and delta K (ΔK) with K allowed to range from 2 to 15. **(B)** Membership co-efficient (Q-value) where each horizontal line represents one wheat line and partitioned into seven sub-populations.

The number of subgroups (K) were estimated based on the rate of change in the log probability of data between successive *K*-values. In the plot of K against ΔK, a break in the slope was observed at *K* = 7 followed by flattening of the curve (Figure 1). This indicated that the cultivars could be divided into seven sub-groups. Group I included post-Green Revolution cultivars selected for irrigated areas, group II included post-Green Revolution cultivars adapted for rainfed areas, group III were landraces and their derivatives, group IV were derivatives of Green-Revolution cultivars, group V included Green Revolution cultivars adapted from CIMMYT, group VI were post-Green Revolution cultivars from CIMMYT, and group VII included elite cultivars with an Inqalab-91 background. Almost 65% of the population showed admixture trend, whereas only 35% originated from a single genetic background. Linkage disequilibrium (LD) analysis performed on all entries indicated the highest LD in the B genome, followed by D and then A genomes. The B genome had the highest LD in chromosome 1B due to the 1B.1R translocation. LD decay rate for the B genome was 25 cM followed by D (20 cM) and A genomes (7 cM). The frequency of physically linked locus pairs in significant LD was 31% overall (*r*^2^ = 0.2), among which the D genome had the highest proportion of marker pairs in LD (41%), followed by B (35%) and A (25%) genome. The estimated average *r*^2^ for unlinked loci was 0.024. Chromosome 6D had the maximum number of marker pair in LD (73%), followed by 2D (48%) and 1B (46%). LD decreased with increasing genetic distance between marker loci. In all three cases, the *r*^2^ declined within 12 cM below critical values. In GWAS, there are two ways to account for confounding effect of population structure: (i) statistically accounting population structure, and (ii) carefully selection of the association mapping panel to reduce the range of phenology. We employed first strategy because the number of cultivars released in Pakistan were not sufficient for further selection based on phenology.

### Marker–trait associations for yield related traits

**Figure 2:**
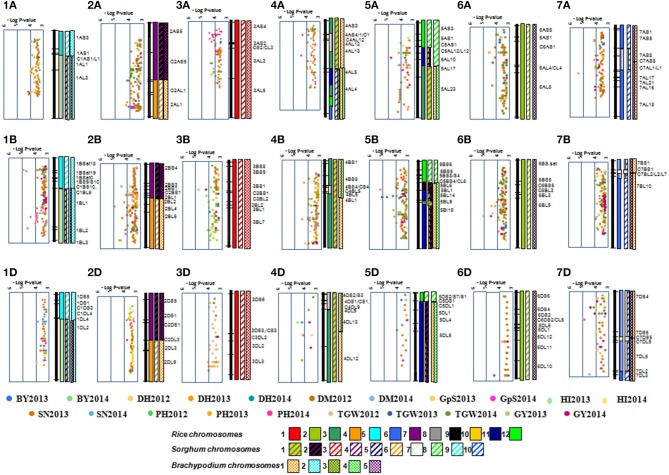
**Distribution of 2203 MTA on wheat chromosomes bin based on their—log *P*-value**. Orthologous chromosome are presented in different color codes.

A total, 2094 MTA were detected at -log *P* >3 using MLM; however, only 44 MTAs with -log P >4 were considered to avoid false positive impact of alleles (Table 3). The highest numbers of MTAs were detected for PH (10) and TGW (10), followed by GY (5), BY (5), DH (5), DM (3), SN (3) and HI (3). The highest numbers of MTAs were identified on chromosomes 2B (6) and 5B (6), followed by 1A (5) and 2A (5), whereas no MTA was found on chromosomes 1D, 2D, 3D, and 5D. The D genome had the lowest number of MTAs (4), whereas the B genome had the highest number (23), followed by the A genome (17). Multi-trait loci were detected on chromosome 1A (BY and GY), 1B (DH and DM), 2B (DH, DM, and PH), 4B (BY and GY), and 6B (HI, BY, and GY). MTAs on chromosomes 5B and 6B were frequently detected for different traits across years. Haplotypes generated for all yield components are compared based on—log *P*-values on bin based synteny maps of wheat, rice, and sorghum (Figure 2).

**Table 3:** **Description of MTAs for yield components in historical Pakistani wheat cultivars from, and their synteny to rice, sorghum and Brachpodium**.

**S. No**.	**Marker name**	**Chromo-some**	**Position**	**Seq. description**	**Trait^a^**	**MLM (-logp)**	**454 contigs**	**Rice pseudo-molecule**	**Annotation rice**	**Sorghum pseudo-molecule**	**Annotation sorghum**	**Brachypodium pseudo-molecule**	**Annotation Brachypodium**	**Associated QTL citation**
1	Kukri_c136_857	1AL	85.36	—NA—	BY_2014	4.25	–	–	–	–	–	–	–	
2	Kukri_c136_857	1AL	85.36	—NA—	GY_2014	4.23	–	–	–	–	–	–	–	
3	Tdurum_contig80278_250	1AL	86.43	Galactosylgalactosylxylosylprotein 3-beta-Glucuronosyltransferase 1	BY_2014	4.21	3841476_1845_33119_166376+,802059-	–	–	Sb09g025880	Transducin family protein/WD-40 repeat family protein	–	–	Chu et al., 2008
4	Tdurum_contig80278_250	1AL	86.43	Galactosylgalactosylxylosylprotein 3-beta-Glucuronosyltransferase 1	GY_2014	4.1	3841476_1845_33119_166376+,802059-	–	–	Sb09g025880	Transducin family protein/WD-40 repeat family protein	–	–	
5	IAAV1930	1AL	104.35	—NA—	SN_2013	4.17	–	–	–	–	–	–	–	Cuthbert et al., 2008
6	Excalibur_c8052_541	1BS	47.41	e3 ubiquitin-protein ligase herc2	DH_2014	5.03	–	–	–	–	–	–	–	
7	Excalibur_c8052_541	1BS	47.41	e3 ubiquitin-protein ligase herc2	DM_2014	5.01	–	–	–	–	–	–	–	
8	Ku_c35823_743	2AL	164.87	—NA—	TGW_2014	4.09	6360901_15771_597154_2428320+,…,3391778-	–	–	–	–	Bradi5g22110	NOI RPM1-interacting protein 4 (RIN4) family protein	
9	CAP11_c2194_115	2AL	164.87	—NA—	TGW_2014	4.07	–	–	–	–	–	–	–	McCartney et al., 2006
10	RAC875_rep_c77617_1454	2AL	164.87	Serine threonine-protein phosphatase 6 Regulatory subunit 3-like isoform x1	TGW_2014	4.2	–	–	–	–	–	–	–	
11	Tdurum_contig56321_232	2AL	191.58	—NA—	PH_2013	4.2	–	–	–	–	–	–	–	
12	BS00027799_51	2AL	192.76	—NA—	PH_2013	4.28	–	–	–	–	–	–	–	
13	Kukri_c51619_265	2BS	57.3	—NA—	DH_2014	4.31	5231691_32301_859367_4542148+,…,66242+	–	–	Sb02g041910	CHR5 (chromatin remodeling 5)	–	–	Chu et al., 2008; Mason et al., 2010
14	Kukri_c51619_265	2BS	57.3	—NA—	DM_2014	4.32	5231691_32301_859367_4542148+,…,66242+	–	–	Sb02g041910	CHR5 (chromatin remodeling 5)	–	–	
15	RAC875_rep_c114945_465	2BS	78.69	Heat shock cognate 70 kda protein 1	PH_2013	4.1	8005258_14430_569572_3812214-,…,669014-	–	–	Sb06g014400	HSP70 (heat shock protein 70)	Bradi1g03720	HSP70-1 heat shock cognate protein	Börner et al., 2002; Groos et al., 2003, 2007
16	RAC875_c36104_356	2BS	79.75	—NA—	DH_2014	4.9	–	–	–	–	–	–	–	
17	Excalibur_c80601_308	2BS	86.13	—NA—	SN_2013	4.26	8013217_9638_240085_4220635+,…,2481921+	–	–	–	–	Bradi1g02960	Transducin family protein/WD-40 repeat family protein	
18	BobWhite_c1105_299	2BL	167.12	Oligopeptide transporter 4	PH_2013	4.49	–	–	–	–	–	–	–	
19	BobWhite_c6300_169	3AL	79.43	—NA—	TGW_2014	4.04	–	–	–	–	–	–	–	Börner et al., 2002; Huang et al., 2004
20	Tdurum_contig15529_135	3AL	79.43	—NA—	TGW_2014	4.16	–	–	–	–	–	–	–	
21	RAC875_c15003_377	3AL	97.34	—NA—	PH_2013	4.1	–	–	–	–	–	–	–	Huang et al., 2004
22	BS00074688_51	3BL	85.66	Unnamed protein product	DH_2013	4.27	–	–	–	–	–	–	–	Huang et al., 2004
23	BS00022025_51	3BL	138.34	Glycosyltransferase-like protein	TGW_2012	4.44	10461386_4636_131369_1939977+,…,8297721+	LOC_Os01g70180	Exostosin family domain containing protein, expressed	–	–	–	–	(Huang et al., 2004; Chu et al., 2008; Mason et al., 2010)
24	BS00073411_51	3BL	194.08	—NA—	HI_2014	4.17	–	–	–	–	–	–	–	
25	BS00074345_51	3BL	194.08	Unnamed protein product	HI_2014	4.17	–	–	–	–	–	–	–	Huang et al., 2004; McCartney et al., 2006
26	Kukri_c77040_87	4AL	111.58	—NA—	PH_2014	4.56	–	–	–	–	–	–	–	Börner et al., 2002; Chu et al., 2008
27	RAC875_c23144_1560	4BL	101.24	Upf0202 protein at1g10490-like	BY_2014	4.64	–	–	–	–	–	–	–	
28	RAC875_c23144_1560	4BL	101.24	Upf0202 protein at1g10490-like	GY_2014	4.03	–	–	–	–	–	–	–	
29	BobWhite_c4264_325	4DS	30.5	—NA—	BY_2014	4.3	2269511_8638_506519_1140818-,195155+,1591649-	–	–	–	–	Bradi4g09070	Proline transporter 1	Huang et al., 2006; McCartney et al., 2006; Wang et al., 2009
30	wsnp_Ra_rep_c75364_72953286	5AS	59.09	Methionyl-trna synthetase	PH_2014	4.56	–	–	–	–	–	–	–	Huang et al., 2004
31	tplb0049a09_1302	5AL	237.47	Cell number regulator 6	SN_2013	4	–	–	–	–	–	–	–	
32	wsnp_Ex_rep_c66651_64962429	5BL	93.59	Loc100284144 isoform x1	TGW_2013	4.21	10903276_18551_463202_9591776-,…,7515457-	LOC_Os09g24710	Alpha/betahydrolase fold, putative,	Sb02g024630	Unknown protein	Bradi4g30210	Alpha/beta-Hydrolases superfamily protein	
33	BS00041704_51	5BL	94.94	—NA—	TGW_2013	5.12	10886391_9955_144073_8253225+,…,3906427-	–	–	–	–	Bradi4g32870	Mmethyl-CPG-binding domain 8	
34	Excalibur_c49271_183	5BL	94.94	—NA—	TGW_2013	5.17	10924151_7441_71843_10763018-,335478+	LOC_Os09g29430	Citratetransporter, putative, expressed	Sb02g027290	Oxoglutarate:malate antiporter	Bradi4g32730	Dicarboxylate transport 2.1	
35	IAAV4074	5BL	94.94	Hypothetical protein F775_08920	TGW_2013	4.73	–	–	–	–	–	–	–	
36	BS00009311_51	5BL	102.5	—NA—	PH_2012	4.17	10919029_7573_89481_10719715+,…,8133780+	–	–	Sb02g000930	Flavin-containing monooxygenase family protein/FMO family protein	–	–	
37	TA002629-0202	5BL	103.24	—NA—	PH_2012	4.09	–	–	–	–	–	–	–	
38	RFL_Contig3175_1271	6AL	141.69	NADH dehydrogenase	PH_2012	4.06	5833640_8654_124173_5725598+,403976+,5730269+	LOC_Os02g57180	NADH dehydrogenase 1 alpha9, mitochondrial	–	–	–	–	Börner et al., 2002
39	wsnp_Ku_rep_c101817_88911480	6BL	83.84	—NA—	HI_2014	4.19	–	–	–	–	–	–	–	
40	Tdurum_contig53125_1716	6BL	90.97	—NA—	BY_2014	4.95	–	–	–	–	–	–	–	Börner et al., 2002
41	Tdurum_contig53125_1716	6BL	90.97	—NA—	GY_2014	4.33	–	–	–	–	–	–	–	Mir et al., 2012; Wang et al., 2011
42	tplb0024a09_742	7DS	50.9	Rna polymerase ii transcription partial	GY_2014	4.14	–	–	–	–	–	–	–	Charmet et al., 2001; Li et al., 2007
43	BS00099499_51	7DL	176.37	Predicted protein	DH_2014	4.21	–	–	–	–	–	–	–	Narasimhamoorthy et al., 2006; Charmet et al., 2001; Sun et al., 2009; Wang et al., 2009
44	BS00099499_51	7DL	176.37	Predicted protein	DM_2014	4.21	–	–	–	–	–	–	–	

a *See the footnote of Table 1 for abbreviations*.

### Gene annotation

Gene ontology (GO) of 44 MTA sequences were assessed using BLAST2GO v.3 software and SNPs were further annotated using the TriAnnot Pipeline. Twenty (54.5%) SNPs were annotated as present within genes (Table 3). The transcripts of these genes encoded binding proteins (3), response to stimulus protein (3), catalytic activity proteins (2), cellular proteins (2), organelle related proteins (1), a single-organism process protein (1), a cellular process protein (1), a metabolic process proteins (1), a membrane protein (1), a reproductive process protein (1), a developmental process protein (1), and a multicellular organismal process protein (1).

### Synteny-based dissection of yield components wheat cultivars

The alignment of traits-associated SNPs to 454-wheat contigs and their synteny to rice, *Brachypodium*, and sorghum aided identification of orthologous regions. Out of 2094 SNPs associated with traits, 540 were aligned against contigs with cut-off values of 70% CIP and 70% CALP. These assembled contigs were aligned with 240 rice, 388 *Brachypodium*, and 210 *Sorghum* annotated genes. However, only 16 out of 44 stable MTAs could be aligned against wheat contig sequences. Seventeen markers co-localized with five annotated genes of rice, nine of sorghum, and 10 of *Brachypodium* (Table 3).

### Effects of favorable alleles on important agronomic traits

**Figure 3:**
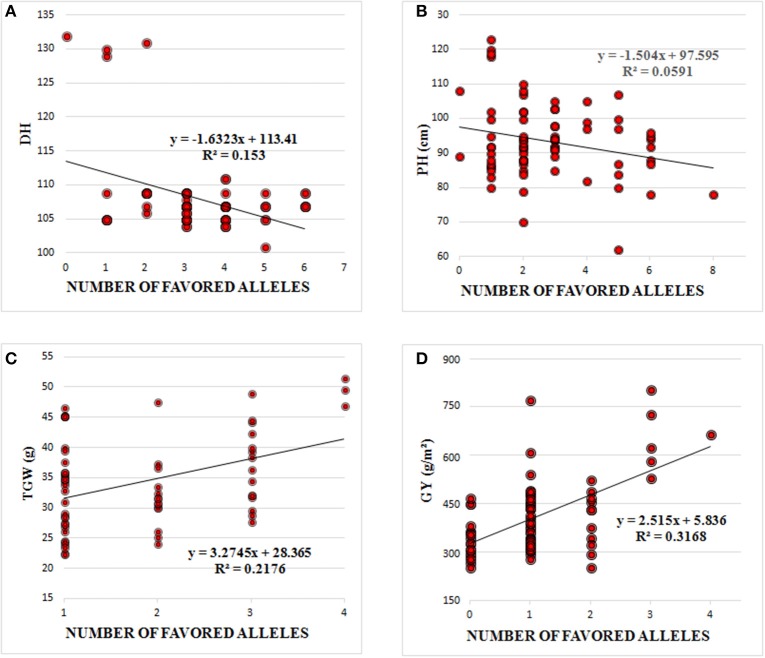
**Linear regression between number of favored alleles and (A) days to heading (DH), (B) Plant Height (PH), (C) Thousand Grain Weight (TGW), and (D) Grain Yield (GY)**.

The favorable alleles of significantly associated SNPs had minor additive effects on DH, PH, TGW, and GY (Figure 3). DH and PH were decreased by increasing the number of favorable alleles and linear regressions (R2) between numbers of favorable alleles and phenotype were 0.15 and 0.06, respectively. Similarly, the higher frequency of favorable alleles of associated SNPs additively enhanced TGW and GY with R2 of 0.22 and 0.32, respectively (Figure 3). There had been no selection pressure on any of these favorable alleles, which were evenly distributed across old and modern cultivars. However, the frequency of favorable alleles for DH, PH, TGW, and GY were slightly higher in cultivars released post-2000, compared to those released before 2000 (Table 3). DH and PH fell from 108.7 to 106.7 days and 94.0 to 91.8 cm, and TGW and GY increased from 32.2 to 36.4 (g) and 422.4 to 521.5 (g m^−2^) in pre-2000 compared to post-2000 cultivars, respectively (Table 4).

**Table 4:** **Percentage frequency of favorable alleles for DH, days to heading; PH, plant height; TGW, thousand grain weight; and GY, grain yield in cultivars released before and after 2000**.

**Trait^a^**	Cultivar group^**^	**Mean**	Frequency (%) of favorable alleles^*^					
			**0**	**1**	**2**	**3**	**4**	**≥5**
DH	Pre-2000	108.73	1.5	8.8	14.7	33.8	26.5	7.4
	Post-2000	106.9	0	4.9	22	39	19.5	12.2
PH	Pre-2000	94.05	2.9	23.5	26.5	25	7.4	7.4
	Post-2000	91.85	0	22	39	14.6	4.9	9.8
TGW	Pre-2000	32.25	13.2	44.1	25	16.2	1.5	0
	Post-2000	36.44	17.1	43.9	22	12.2	4.9	0
GY	Pre-2000	422.4	23.5	57.4	13.2	5.9	0	0
	Post-2000	521.5	34.1	43.9	9.8	7.3	4.9	0

* *Frequency refers to number of cultivars with 0, 1, 2 3, 4, and 5 favorable alleles*.

** *Cultivar groups refers to 68 cultivars released before year 2000 (pre-2000) and 41 cultivars released after 2000 (post-2000)*.

a *See the footnote of Table 1 for abbreviations*.

## Discussion

### Genetic and phenotypic structure of historical wheat cultivars

The significant proportion of wheat cultivars from Pakistan are derived from CIMMYT germplasm, either as directly released cultivars, or as parents in crossing programs that led to new cultivars. Population structure provided the confirmatory evidence that CIMMYT cultivars like Mexipak-65, Pavon76 and SeriM82 (syn. Pak-81) appeared in same sub-populations along with Pakistani cultivars. Secondly, the population structure clearly dissected the cultivars released for irrigated and rainfed environments, indicating different genetic backgrounds used in developing cultivars for these two regions. 1BL.1RS translocation lines from CIMMYT are important breeding source which adapted very well in rainfed environments of Pakistan. Earlier studies reported that 1BL.1RS lines had higher root and shoot dry weight under drought treatments and an increased root/shoot ratio (Hoffmann, 2008). In addition, research has shown increased efficiencies of light conversion, particularly with the 1BL.1RS translocation, hence biomass was increased. The 1BL.1RS translocation has been used intensively in wheat breeding, but less at CIMMYT in recent times, due to its negative effect on quality (Sukumaran et al., 2015). However, recent report indicated the presence of 1B.1R translocation in cultivars released recently in Pakistan (Rasheed et al., 2015). Population structure based on SNP markers also identified the breeding activities independent of CIMMYT germplasm by clustering cultivars in group VII. This group clustered together the Inqalab-91, a leading, and pre-dominant cultivar, and its derivatives. Another important finding is the separate clustering of landraces, indicating the limited use of landraces in recent breeding in Pakistan, in agreement with our recent findings based on analysis of functional genes (Rasheed et al., 2015).

Phenotypic characterization for yield-related traits indicated that historical germplasm has significant variation in traits like TGW, GpS, and SN (Table 1). Therefore, genetic dissection of important economic traits in historical cultivars could be useful for utilization in wheat genetic improvement. Nine traits selected to dissect yield and some were positively correlated with GY as previously reported (Dodig et al., 2012; Sukumaran et al., 2015). Our results on LD using a 90K SNP assay were partially different with previous reports. LD was higher in the B and D genomes than in the A genome. This was largely due to selection of *Glu-B1* alleles that are important for chapatti making, and frequent presence of the 1B.1R translocation in cultivars grown after 1981 (Rasheed et al., 2015). However, higher LD involving SNPs in the D genome is in agreement with previous studies and is due to limited infusion of *A. tauschii* germplasm in the evolution of bread wheat (Wang et al., 2013).

### Marker-trait associations

The ultimate breeding objective is the higher gains for GY, a very complex trait with low heritability. In contrast, yield components like thousand kernel weight (not spike number) are more stable across environments and have higher heritabilities (Lopes et al., 2014). Therefore, identifying stable QTL for yield components having higher correlation with GY can be helpful to manipulate GY. In this study, MTAs involving GY and related traits were identified on all wheat chromosomes, including consistent and environmentally specific MTAs. They are compared to MTAs identified in other studies (Table 3 and Supplementary Data Sheet 1), and 18 MTAs co-localized with QTL identified in earlier studies. It is important to mention that phenotypic trials were conducted in rainfed environment, therefore MTAs identified in this study are important because they may be linked to minor genes adaptation to rainfed environments.

MTAs for DH were identified on chromosome 1BS, 2BL, 2BS, and 7DL. Those present on 1BS, 2BS, and 7DL also had pleitropic effect on DM. The MTA for DH and DM on chromosome 2BS corresponded to a previously identified chromosomal region (Chu et al., 2008; Mason et al., 2010). Similarly, the MTA linked to DH and DM on 7DL was reported earlier in biparental QTL analyses in several studies (Charmet et al., 2001; Narasimhamoorthy et al., 2006; Wang et al., 2009). PH is another important trait to deploy especially in rainfed environments. Semi-dwarf cultivars were major contributors to the success of the Green Revolution in the Indian sub-continent. Stable MTAs linked to PH on chromosomes 2AL, 2BL, 3AL, 4AL, 5AS, 5BL, and 6AL, were identified in the current study. An MTA for PH on chromosome 2BL was within confidence intervals of previously identified QTL (Börner et al., 2002; Groos et al., 2003, 2007). Similarly, MTAs on chromosome 5AS and 6AL are most likely the QTL reported earlier (Börner et al., 2002; Huang et al., 2004; Sukumaran et al., 2015). The MTA for PH on chromosome 3A corresponded to *OsGA2ox3* (Sakamoto et al., 2004), the 4A MTA corresponded to an *ent*-kaurenic acid oxidase (KAO) gene in the gibberellin metabolic pathway affecting PH (Khlestkina et al., 2010), and a chromosome 5A MTA corresponded to the *Rht9* locus (Ellis et al., 2005). Although *Rht-B1* and *Rht-D1* on chromosomes 4B and 4D were significantly associated with PH in historical Pakistani cultivars (Rasheed et al., 2015), we found no MTAs on chromosomes 4B and 4D indicated SNPs linked to these genes were either not present in 90K assay or were excluded during quality control.

MTAs for TGW were present on chromosomes 2AL, 3AL, 3BL, and 5BL. MTA on 5BL had the pleitropic effect on PH and TGW, and can be important target to manipulate both traits simultaneously. Chromosome 2AL had three MTAs at the same position within a region previously reported for a biparntal population (McCartney et al., 2006). Similarly, MTAs for SN were present on 1A, 2B, and 5A chromosomes. Previously, stable genomic regions on chromosomes 3B, 5A, and 5B have been implicated in yield and related traits (Pinto et al., 2010; Edae et al., 2014; Lopes et al., 2014). In CIMMYT germplasm, they are perhaps most important genomic regions associated with multiple yield related traits (Sukumaran et al., 2015). This is important to mention that TGW and grain number are the primary components of GY, and are usually negatively correlated in segregating populations. Therefore, it has been important and challenging strategy to identify the genomic regions enhancing TGW without significant reduction in grain number (Griffiths et al., 2015). In our study, the MTA associated with these two traits were independent of each other, however their allelic effects were minor, and the extent of trade-offs between grain numbers and TGW may be reduced. Although genetic gain for yield has been achieved by increasing grain number, however it is possible to produce high yielding cultivars with large grains. For example, CIMMYT cultivars Bavicora and Kambara have relatively large grains (Sayre et al., 1997). This has led to investigate the underlying genes controlling grain size and weight, and several genes have been documented recently (Valluru et al., 2014). The analysis of such functional genes for grain size and weight in same germplasm indicated that *TaSus2-2B, TaGW2-6A*, and *TaGS-D1* have larger effects on TGW as compared to *TaCwi-2A* and *TaCKX-D1* (Rasheed et al., 2015). These functional genes and SNPs associated with TGW in this study can help in designing new strategies to accumulate favorable alleles for TGW in future wheat breeding.

MTA responsible for GY were identified on chromosomes 1AL, 4BL, 6BL, and 7DL, and only 7DL MTA was reported in biparental QTL analyses (Charmet et al., 2001; Bossolini et al., 2007). MTAs associated with multiple traits and referred as pleiotropic MTAs were identified for GY and BY on chromosomes 1AL, 4BL, and 6BL. Same SNP marker was associated with GY and BY on chromosome 6BL at 90.9 cM, however, another MTA was identified for HI on chromosome 6BL at 83.8 cM. Given the LD decay of 8–10 cM these regions are in strong LD, and they may be controlled by the same locus.

### Annotation and synteny of trait associated SNP

Cloning and gene identification in wheat is a difficult task, but is becoming less daunting due to the extensive genomics efforts by the international wheat community. Although comparative genomics is a powerful tool in trait dissection, confirmation of the roles of individual genes will still be needed through functional validation. The large rainfed Indo-Pakistan plain is undergoing climate change. As mentioned above, the genomic regions identified in this study could be fruitful for future breeding programs due to their role in combating stresses created by climate change. Annotation of falking sequences and their syntenic relationship to the genes in rice, sorgum and Brachypodium confirmed our findings that these genomic regions encode proteins which are important components of pathways triggered in response to stressed environments. For example, aldehyde dehydrogenase (Yu et al., 2014), cell number regulator six protein (Guo et al., 2010), glycosyltransferase-like protein (Wang et al., 2015), molybdenum cofactor sulfurase (Lu et al., 2013), n-acetylglucosaminyltransferase III (Nguema-Ona et al., 2014), NADH dehydrogenase, and serine threonine-protein phosphatase six (Mao et al., 2010; Zhang et al., 2010a) were reported in earlier studies to be linked to plant metabolic stability. These genes are present in genomic regions associated with yield traits and can be considered possible candidate genes for yield improvement as well as for future cloning of these loci.

### Allelic effects of trait associated SNPs

The overall superiority of post-2000 cultivars highlights the considerable progress made during the past decade, and the significant improvement achieved for important economic traits like DH, PH, TGW, and GY. There were minor additive relationships between numbers of favorable alleles and agronomic traits, indicating that genetic improvement of these traits were independent and therefore can be combined. Many of the identified MTAs seem to involve genes that are components of networks driven by the prevailing environmental plasticity and show little improvement in phenotypic stability. The results are also interesting in that none of the favorable alleles has been a breeding target during cutlivar development. This is in contrast to favorable alleles of functional genes for adaptability (*Ppd-D1, Rht-B1*, and *Rht-D1*) and TGW (*TaGW2-6A* and *TaCKX6-D1*) deployed in Pakistani wheat cultivars (Rasheed et al., 2015). In this situation, cultivars with maximum numbers of favorable alleles can be used as breeding resources and the present results may provide a basis to initiate genomic selection methods (Jannink, 2007) to maximize genetic gains.

## Conclusion

GWAS identified chromosomal regions associated with yield traits in historical germplasm deployed in Pakistan. Annotation and comparative genomics assigned 14 of those MTAs to different metabolic functions which have implications to combat stressed environments. These MTAs can be important targets for marker-based breeding and fine mapping of functional genes after further validation.

### Conflict of interest statement

The authors declare that the research was conducted in the absence of any commercial or financial relationships that could be construed as a potential conflict of interest.

## Supplementary material

Supplementary Figure 1 **Frequency distribution, correlation, and scatter plot of mean values of nine yield components in historical wheat cultivars Days to heading (DH), days to maturity (DM), plant height (PH), grains per spike (GpS), spike density (SN), thousand grain weight (TGW), grain yield (GY), biological yield (BY), and harvest index (HI)**.

Supplementary Table 1 **Passport data of historical set of Pakistan decrypting for each cultivar; its synonym, release year, released by, CIMMYT Br Line, plant height, pedigree and selection History**.

Supplementary Data Sheet 1 **Description of annotation and function of annotated MTAs**.
