# Supplementary figures and images for: Genome-wide association for grain yield under rainfed conditions in historical wheat cultivars from Pakistan
Source: Front Plant Sci. 2015 Sep 22;6:743. doi: 10.3389/fpls.2015.00743 (PMC4585131; doi:10.3389/fpls.2015.00743)

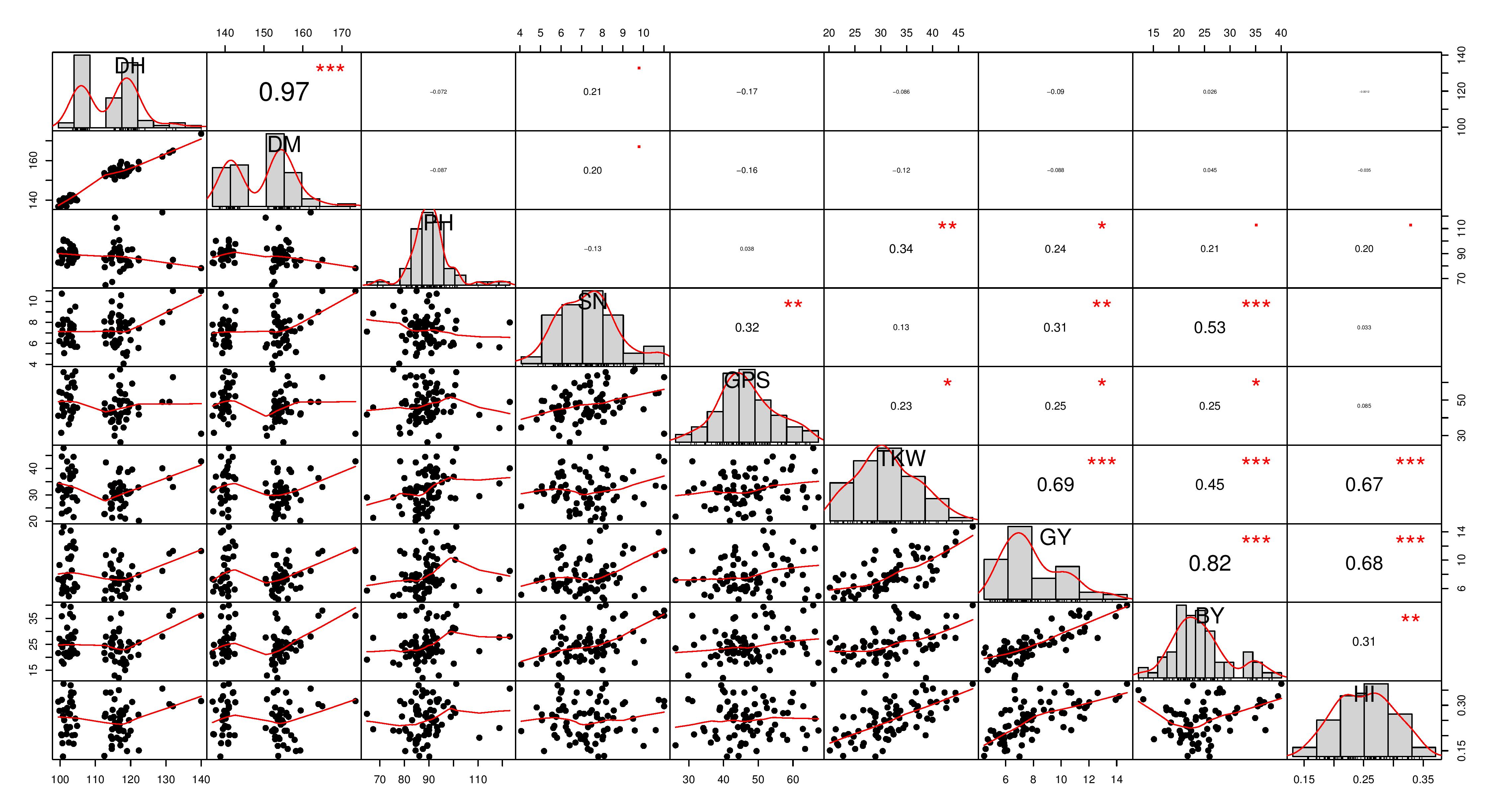

Supplement: Supplementary Figure 1 — Frequency distribution, correlation, and scatter plot of mean values of nine yield components in historical wheat cultivars Days to heading (DH), days to maturity (DM), plant height (PH), grains per spike (GpS), spike density (SN), thousand grain weight (TGW), grain yield (GY), biological yield (BY), and harvest index (HI). [file Image1.JPEG]
