# Supplementary material for: Genome-wide association for grain yield under rainfed conditions in historical wheat cultivars from Pakistan
Source: Front Plant Sci. 2015 Sep 22;6:743. doi: 10.3389/fpls.2015.00743 (PMC4585131; doi:10.3389/fpls.2015.00743)
Supplement: Supplementary Data Sheet 1 — Description of annotation and function of annotated MTAs. [file DataSheet1.DOCX]

**Supplementary Data Sheet for: Genome-wide association for grain yield under semi-arid conditions in historical wheat cultivars from Pakistan**

Qurat-ul-Ain^1^, Awais Rasheed^2,5^, Alia Anwar^1^, Tariq Mahmood^3^, Muhammad Imtiaz^4^, Tariq Mahmood^1^, Xianchun Xia^2^, **Zhonghu He**^2,5^, Umar Masood Quraishi^1^

**^1^Quaid-i-Azam University,** Department of Plant Sciences, Islamabad 45320, Pakistan

**^2^Chinese Academy of Agricultural Sciences (CAAS)**, Institute of Crop Science, National Wheat Improvement Cente, 12 Zhongguancun South Street, Beijing 100081, China

**^3^Higher Education Commission,** Resrach and Development, H-8, Islamabad , Pakistan.

**^4^International Maize and Wheat Improvement Center (CIMMYT)**, Pakistan office, c/o National Agriculture Research Center (NARC), Islamabad 100081, Pakistan

**^5^International Maize and Wheat Improvement Center (CIMMYT)**, c/o CAAS,
12 Zhongguancun South Street, Beijing 100081, China

**Corresponding author**: Umar Masood Quraishi^1^ Email: umasood@qau.edu.pk

### Functions of MTAs

The MTAs associated with BY identified on chromosome 1AL, 4DS and 6BL appeared to be same identified previously ([Chu et al., 2008](#_ENREF_4)), while MTA on chromosomes 4BL was not identified previously and appeared to be novel. The sequence analysis of SNP on 1AL associated with BY revealed the presence of gene 3-beta-glucuronosyltransferase 1 implicated in metabolic process, transferase activity, methionyl-trna synthetase for nucleotide binding, RNA binding and is orthologous to the transducin family protein / WD-40 repeat family protein on chromosome 9 of sorghum. MTA on 4BL was annotated which translate upf0202 protein at1g10490-like, which initiates the metabolic process and N-acetyltransferase activity. MTA on 4DS linked to BY was also reported earlier in biparental QTL analysis for BY ([McCartney et al., 2005](#_ENREF_13);[Huang et al., 2006](#_ENREF_9);[Wang et al., 2009](#_ENREF_18)), and has an orthologous relationship with proline transporter 1 on chromosome 4 of *Brachypodium*. In rainfed environment, proline plays a vital role in drought tolerance and yield stability ([Nezhadahmadi et al., 2013](#_ENREF_16)). MTA on 6BL was reported earlier for BY in biparental QTL analysis ([Borner et al., 2002a](#_ENREF_5)).

The MTA linked to DM were identified on chromosome 1BS, 2BS, and 7DL. The flanking sequence analysis of linked SNP on 2BS MTA showed that it is orthologous to the CHR5 (chromatin remodeling 5); ATP binding / DNA binding / chromatin binding / helicase/ nucleic acid on chromosome 2 of sorghum. The MTA identified for DH were identified on chromosome 1BS, 2BS, 2BL, and 7DL. MTA on chromosome 2BS corresponds to previously identified chromosomal region DH ([Chu et al., 2008](#_ENREF_4);[Esten Mason et al., 2010](#_ENREF_6)). Similarly, sequence analysis of 1BS MTA revealed that SNP is ortholog to e3 ubiquitin-protein ligase herc2 implicated in for retention of repair proteins on damaged chromosomes. MTA on 7DL linked to DH have been reported earlier in biparental QTL analysis ([Charmet et al., 2001](#_ENREF_3);[Narasimhamoorthy et al., 2006](#_ENREF_15);[Wang et al., 2009](#_ENREF_18)).

PH is one of most important trait to deploy in given environmental plasticity. The release of semi-dwarf wheat cultivars originated from CIMMYT were the major driving force in the success of so called Green-revolution in Pak-Indo gigantic plain. Stable MTAs linked to PH on chromosomes 2AL, 2BL, 3AL, 4AL, 5AS, 5BL, and 6AL were identified in our study. MTA on chromosome 2BL was present inside confidence interval of previously identified QTLs for PH ([Borner et al., 2002](#_ENREF_1);[Groos et al., 2003](#_ENREF_8);[Groos et al., 2007](#_ENREF_7)). Annotation of sequence revealed that the SNP is present on heat shock cognate 70 kda protein 1 which generates response against viruses, and is orthologous to the HSP70 (heat shock protein 70) on chromosome 6 of sorghum and chromosome 1 of *Brachypodium*. MTA on 6AL have been reported earlier ([Borner et al., 2002](#_ENREF_1)), having annotation linked to NADH dehydrogenase, which regulates the electron transport chain and respiration, has an orthologous relationship with NADH dehydrogenase 1 alpha 9 on chromosome 2 of rice. The MTA on 5AS was reported earlier for yield in biparental QTL analysis ([Huang et al., 2004](#_ENREF_10)), and is annotated to be methionyl-tRNA-synthetase and is involved in ATP/ tRNA binding; generates response to cadmium ion; protein phosphorylation. Moreover, MTA for PH on chromosome 3A corresponded to mapped gene *OsGA2ox3*;([Sakamoto et al., 2004](#_ENREF_17)), 4A MTA corresponded to *ent*-kaurenic acid oxidase (KAO) gene of gibberellin metabolic pathway gene affecting PH ([Khlestkina et al., 2010](#_ENREF_11))}, and 5A MTA corrosponded to *Rht-9* locus ([Ellis et al., 2005](#_ENREF_5)).

The only MTA associated to SL was present on chromosomes 6AL and annotated to be hypothetical protein, TRIUR3_26908, which is present on contig with TLD-domain containing nucleolar protein orthologous to gene on chromosome 3 of *Brachypodium.*

The MTA for TNo were present on chromosomes 2AL, 3AS, 6AL and 6BL. Sequence analysis of 6AL MTA revealed that SNP is linked to hypothetical protein, TRIUR3_18540, implicated in protein import into chloroplast stroma and is orthologous to the TIC 20 translocon at the inner envelope membrane of chloroplasts 20 on chromosome 2, 4, and 3 of Rice, sorghum, and *Brachypodium,* respectively. MTAs on 6AL and 6BL have been previosuly identified as QTL for GY ([Borner et al., 2002](#_ENREF_1);[Huang et al., 2004](#_ENREF_10)).

The MTAs linked to SpPS were identified on chromosome 1BS, 1BL, 5BL, 7AL, 7BL and 7DL. MTA identified on chromosome 1BS is present under previously reported QTL for GY ([Huang et al., 2004](#_ENREF_10);[McCartney et al., 2005](#_ENREF_13)). 1BS MTA is presnt on 454-contig of wheat carrying hypothetical expressed protein with orthologous relation with hypothetical express protein gene on chromosome 2 of *Brachypodium.* MTA on 5BL has been reported earlier in many studies ([Groos et al., 2003](#_ENREF_8);[Huang et al., 2004](#_ENREF_10);[Groos et al., 2007](#_ENREF_7)) and underpin molybdenum cofactor sulfurase having catalytic activity and pyridoxal phosphate binding functions. Whereas, MTA on chromosome 7BL is annotated to be aldehyde dehydrogenase family protein linked to various important metabolic functions *i.e.* oxidation-reduction process; ATP binding; response to cadmium ion; vegetative to reproductive phase transition of meristem. This MTA has been previously reported and is associated to grain numbers in biparental QTL analysis ([Borner et al., 2002](#_ENREF_1)). However, MTAs on 1BL, 7AL and 7DL were not reported in earlier studies and are non geneic.

The MTAs for TGW were present on chromosomes 2AL, 3AL, 3BL and 5BL. Chromosome 2AL had three MTAs present on same position, which were within the region previously reported for TGW in bipernatal population ([McCartney et al., 2006](#_ENREF_14)). One of the MTA was present on Serine threonine-protein phosphatase-6 regulatory subunit, responsible of multiple stress tolerances in Arabidopsis ([Mao et al., 2010](#_ENREF_12);[Zhang et al., 2010](#_ENREF_19)). The corresponding wheat 454-contig is othologous to chromosome 5 of *Brachypodium* with gene NOI RPM1-interacting protein 4 (RIN4) family protein. The sequence analysis of 3BL MTA revealed that SNP arthologous to glycosyltransferase-like protein responsible for activating secondary cell wall biogenesis; transferase activity; glucuronoxylan biosynthetic, and is present on 454-contig containing orthologous exostosin family domain containing protein on chromosome 1 of rice. MTA on 5BL may be novel which was annotated to LOC100284144 isoform, which intiates the zinc ion binding. This 5BL MTA was present on contig with an orthologous relationship to alpha/beta hydrolase fold putative gene on chromosome 9, 2 and 4 of rice, sorghum and *Brachypodium* respectivly, whereas second MTA on 5BL was present on contig having orthologous genes; citrate transporter putative expressed on chromosome 2 of rice, oxoglutarate malate antiporter on chromsome 2 of sorghum and dicarboxylate transport 2.1 on chromsome 4 of *Brachypodium*.

The MTA responsible for GY were identified on chromosome 1AL, 4BL, 6BL, and 7DL. Galactosyl galactosyl xylosyl protein 3-beta-glucuronosyl transferase 1 was annotation for MTA on chromosome 1AL involved in transferase activity, metabolic process and also had an orthologus relation with transducin family protein / WD-40 repeat family protein on chromsome 9 of *Sorghum*. Another MTA for GY on chromosome 7DL underpin RNA polymerase ii transcription partial for the regulation of transcription from RNA polymerase II promoter, and this QTL has been reported earlier in biparental QTL analysis ([Charmet et al., 2001](#_ENREF_3);[Bossolini et al., 2007](#_ENREF_2)).

Borner, A., Schumann, E., Furste, A., Coster, H., Leithold, B., Roder, M., and Weber, W. (2002). Mapping of quantitative trait loci for agronomic important characters in hexaploid wheat(Triticum aestivumL.). *Theor Appl Genet* 105**,** 921 - 936.

Bossolini, E., Wicker, T., Knobel, P.A., and Keller, B. (2007). Comparison of orthologous loci from small grass genomes Brachypodium and rice: implications for wheat genomics and grass genome annotation. *Plant J* 49**,** 704-717.

Charmet, G., Robert, N., Perretant, M., Gay, G., Sourdille, P., Groos, C., Bernard, S., and Bernard, M. (2001). "Marker assisted recurrent selection for cumulating QTLs for bread-making related traits," in *Wheat in a Global Environment*. Springer), 211-217.

Chu, C.G., Xu, S.S., Friesen, T.L., and Faris, J.D. (2008). Whole genome mapping in a wheat doubled haploid population using SSRs and TRAPs and the identification of QTL for agronomic traits. *Molecular Breeding* 22**,** 251-266. doi: 10.1007/s11032-008-9171-9.

Ellis, M.H., Rebetzke, G.J., Azanza, F., Richards, R.A., and Spielmeyer, W. (2005). Molecular mapping of gibberellin-responsive dwarfing genes in bread wheat. *Theor Appl Genet* 111**,** 423-430. doi: 10.1007/s00122-005-2008-6.

Esten Mason, R., Mondal, S., Beecher, F.W., Pacheco, A., Jampala, B., Ibrahim, A.M.H., and Hays, D.B. (2010). QTL associated with heat susceptibility index in wheat (Triticum aestivum L.) under short-term reproductive stage heat stress. *Euphytica* 174**,** 423-436. doi: 10.1007/s10681-010-0151-x.

Groos, C., Bervas, E., Chanliaud, E., and Charmet, G. (2007). Genetic analysis of bread-making quality scores in bread wheat using a recombinant inbred line population. *Theoretical and Applied Genetics* 115**,** 313-323.

Groos, C., Robert, N., Bervas, E., and Charmet, G. (2003). Genetic analysis of grain protein-content, grain yield and thousand-kernel weight in bread wheat. *Theoretical and Applied Genetics* 106**,** 1032-1040. doi: 10.1007/s00122-002-1111-1.

Huang, X.Q., Cloutier, S., Lycar, L., Radovanovic, N., Humphreys, D.G., Noll, J.S., Somers, D.J., and Brown, P.D. (2006). Molecular detection of QTLs for agronomic and quality traits in a doubled haploid population derived from two Canadian wheats (Triticum aestivum L.). *Theoretical and Applied Genetics* 113**,** 753-766. doi: 10.1007/s00122-006-0346-7.

Huang, X.Q., Kempf, H., Ganal, M.W., and Roder, M.S. (2004). Advanced backcross QTL analysis in progenies derived from a cross between a German elite winter wheat variety and a synthetic wheat (Triticum aestivumL.). *Theoretical and Applied Genetics* 109**,** 933-943. doi: 10.1007/s00122-004-1708-7.

Khlestkina, E.K., Kumar, U., and Röder, M.S. (2010). Ent-kaurenoic acid oxidase genes in wheat. *Molecular breeding* 25**,** 251-258.

Mao, X., Zhang, H., Tian, S., Chang, X., and Jing, R. (2010). TaSnRK2.4, an SNF1-type serine/threonine protein kinase of wheat (Triticum aestivum L.), confers enhanced multistress tolerance in Arabidopsis. *Journal of Experimental Botany* 61**,** 683-696. doi: 10.1093/jxb/erp331.

Mccartney, C.A., Somers, D.J., Humphreys, D.G., Lukow, O., Ames, N., Noll, J., Cloutier, S., and Mccallum, B.D. (2005). Mapping quantitative trait loci controlling agronomic traits in the spring wheat cross RL4452 x 'AC Domain'. *Genome* 48**,** 870-883.

Mccartney, C.A., Somers, D.J., Lukow, O., Ames, N., Noll, J., Cloutier, S., Humphreys, D.G., and Mccallum, B.D. (2006). QTL analysis of quality traits in the spring wheat cross RL4452 x 'AC Domain'. *Plant Breeding* 125**,** 565-575.

Narasimhamoorthy, B., Gill, B.S., Fritz, A.K., Nelson, J.C., and Brown-Guedira, G.L. (2006). Advanced backcross QTL analysis of a hard winter wheat x synthetic wheat population. *Theoretical and Applied Genetics* 112**,** 787-796. doi: DOI 10.1007/s00122-005-0159-0.

Nezhadahmadi, A., Prodhan, Z.H., and Faruq, G. (2013). Drought tolerance in wheat. *ScientificWorldJournal* 2013**,** 610721. doi: 10.1155/2013/610721.

Sakamoto, T., Miura, K., Itoh, H., Tatsumi, T., Ueguchi-Tanaka, M., Ishiyama, K., Kobayashi, M., Agrawal, G.K., Takeda, S., Abe, K., Miyao, A., Hirochika, H., Kitano, H., Ashikari, M., and Matsuoka, M. (2004). An overview of gibberellin metabolism enzyme genes and their related mutants in rice. *Plant Physiol* 134**,** 1642-1653. doi: 10.1104/pp.103.033696.

Wang, R.X., Hai, L., Zhang, X.Y., You, G.X., Yan, C.S., and Xiao, S.H. (2009). QTL mapping for grain filling rate and yield-related traits in RILs of the Chinese winter wheat population Heshangmai X Yu8679. *Theoretical and Applied Genetics* 118**,** 313-325. doi: DOI 10.1007/s00122-008-0901-5.

Zhang, H., Mao, X., Wang, C., and Jing, R. (2010). Overexpression of a common wheat gene TaSnRK2. 8 enhances tolerance to drought, salt and low temperature in Arabidopsis. *PLoS One* 5**,** e16041.
